# Molecular docking analysis of stevioside with Akt and PPARγ

**DOI:** 10.6026/97320630017283

**Published:** 2021-01-31

**Authors:** Abilasha Deenadayalan, S Vijayalakshmi, CS Janaki, Selvaraj Jayaraman

**Affiliations:** 1 Department of Anatomy, Saveetha Medical College and Hospital, Saveetha Institute of Medical and Technical Sciences, Saveetha Nagar,Chennai-602105, India; 2 Department of Anatomy, Asan Memorial Dental College and Hospitals, Asan Nagar, Chengalpattu, Chennai-602 105, India;; 3 Department of Anatomy, Bharath Medical College and Hospital, Selaiyur, Chennai-600 073, India; 4 Department of Biochemistry, Saveetha Dental College and Hospitals, Saveetha Institute of Medical and Technical Sciences, Saveetha University, Chennai-600 077, India

**Keywords:** Diabetes, AKT & PPAR gamma, stevioside, molecular docking

## Abstract

Stevioside is a diterpenoid glycoside consisting of an aglycone (steviol) and three glucose molecules. It is commonly used as an anti-hyperglycemic food because of its non-caloric property. Therefore, it is of interest to document the interactions of stevioside with AKT & PPAR-γ proteins using Autodock Vina PyRx docking techniques. Results of the docking studies indicate that stevioside had more than two hydrogen bond interactions with the AKT and PPAR γ protein for further consideration.

## Background

Diabetes is widely recognized as one of the leading causes of death in the world [1]. High-fat (HFD) and high-sucrose diets (HSD) reduce insulin action of glucose production in vivo, alters gluconeogenesis by increase glucose-6-phosphatase (G-6-Pase) activity in whole cell homogenates [2]. This diet induced metabolic environment triggers oxidative stress result in through the production of free radicals via glucose auto-oxidation,increase ROS and autophagy have a great role in the initiation and progression of diabetes and its complications [3]. Despite these factors, a number of genes and/or receptors involved in metabolism, inflammation, oxidative stress, substrate transport, protein synthesis and alteration, and transcriptional regulation are altered by the chronic intake of HFD. In response to dietary composition, a better understanding of tissue-specific effects would provide guidance on the most suitable HFD- induced animal model(s) to research T2DM pathogenesis and related phenotypic changes [4,5]. Nowadays, there is a great interest in using the medicinal plants and its products for treatment of diabetes. Stevia rebaudiana also known as sweet herb, sweet leaf, honey leaf, candy leaf and honey yerba [6]. From the past few decades, the study of structural, chemical and functional aspects of S. rebaudiana has been in the limelight in order to discover different diterpene glycosides (DGs). Extensive research has led to the identification of more than 30 DGs from S. rebaudiana [7]. Among all the reported DGs, stevioside is most common with properties like slightly bitter taste, non-fermentable, and low calorific value [8]. However, stevioside is 300 folds sugary with extended shelf life as compared to normal sugar [9]. Stevioside, a diterpenoid glycoside comprising an aglycone (steviol) and three molecules of glucose, is widely used as an anti-hyperglycemic food in the world since its non-caloric property, including the European Union and the United States [10]. Stevioside and other sweet steviol glycosides, such as steviobioside, rebaudioside (A-F) and ducoside A, are not absorbed and degraded in the intestine due to the lack of corresponding enzymes in humans; but intestinal bacterial flora can convert steviosides into the final metabolites-steviol and glucose, the resulting glucose is consumed by colon bacteria and is not absorbed into systemic circulation [11]. Studies have showed that steviol glycosides exhibit many therapeutic benefits, such as hypoglycemic, anti-hypertensive, anti-inflammatory, and so on [12].It has been reported that stevioside may enhance insulin secretion which regulates glucose metabolism, leading in reduction of blood pressure and acts as an anti-hyperglycaemic agents [13].Therefore, this study was designed to identify the mechanism of stevioside against diabetics proteins by using molecular docking analysis.

## Methodology

### Preparation of protein:

The AKT (PDB ID: 3QKM) and PPR gamma (PDB ID: 2PRG) atomic coordinates have been retrieved from the RCSB PDB (protein data bank) database, charge assignment, solution measurements and fragment volumes to the protein have been performed using Autodock Tool 4 (ADT) prior to analysis or docking. The protein molecule was then optimized for molecular docking by the Autodock Tool.

### Preparation of ligand:

Stevioside's 2D structure has been retrieved from the pubchem database and translated into the.pdb file format by using online smile translator. In order to enhance the analysis, ligand was first standardized and converted into PDBQT format using the PyRx Virtual Screening Tool (python prescription 0.8) graphical user interface version.

### Molecular docking:

AutoDock (V. 4.0) was used in the PyRx Interface to validate the binding capabilities of the interactions between Stevioside and AKT & PPAR gamma [14,15]. During the docking phase, ligand was thought to be flexible and the protein was considered to be rigid. Using the Pyrex Auto Grid engine generated the grid configuration file. The implementation was also used to know/predict the amino acids that come in contact with ligands at the active protein site. Root-mean-quarter deviation (RMSD) values with less than 1.0Å were considered to be optimal. They are grouped together to find an acceptable binding. The highest binding energy (most negative) was found to be a ligand with high binding affinity.

## Results:

### Molecular Docking interaction of Stevioside with AKT:

**Figure 1:**
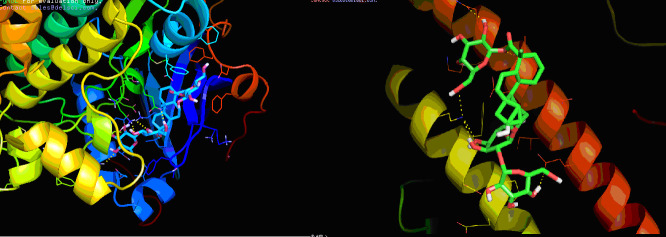
Molecular interaction of stevioside with (a) AKT and (b) PPAR gamma

Stevioside is a particular high-affinity AKT protein compound. The results of the docking analysis shown in Figure 1 clearly indicate that Stevioside had shown binding to AKT protein via hydrogen bonding interactions. Details of drug - receptor interaction were provided in Table 1(see PDF). As illustrated in Table 1(see PDF), stevioside has demonstrated binding energy of-9.6 kcal / mol AKT protein. Three hydrogen bonds have been formed at HIS-134, LYS-276, GLU-278 when stevioside has been docked with AKT.

### Molecular Docking interaction of Stevioside with PPAR gamma:

The results of docking studies indicate that Stevioside with PPAR gamma complex had the least binding affinity of-6.5 Kcal / mol. The least binding affinity suggests that Stevioside forms a stable complex with PPAR gamma. This also forms four hydrogen interactions with the amino acids LYS-373, GLN-437, THR-440 and GLU-448. Details of hydrogen bond interactions are shown in Figure 1 & Table 1(see PDF).

## Discussion:

Interactions among Stevioside and AKT & PPAR gamma proteins were explored to evaluate potential binding affinity using Autodock Vina PyRx docking techniques. The interaction between the two docking complexes has been contrasted on the basis of their binding strength, which in turn involves interaction of hydrogen bonding and other bonding between proteins and ligands. The binding energy provided by the interaction between Stevioside and AKT & PPAR gamma proteins was summarized in Table 1(see PDF). Daisy et al. stated in 2012 that a minimum docking energy has been needed for the formation of a ligand-receptor complex such that the ligand has been buried in the receptor cavity. According to Daisy et al. 2012, Stevioside has been bound to the cavity of AKT & PPAR gamma since it has a minimum docking energy of-9.6 kcal / mol with AKT and-6.5 kcal / mol with PPAR gamma protein. The participation of H-bond interactions enabled the complex to obtain the accepted configuration of the complex structure [16]. Analysis of the docking studies indicates that stevioside had more than two hydrogen bond interactions with the AKT & PPAR gamma protein, indicating that more H-bonds indicated a high affinity of ligand to the receptor.

## Conclusion

We show the molecular docking analysis of stevioside with targets AKT and PPAR gamma in the context of diabetics.
